# The Role of Walkers’ Needs and Expectations in Supporting Maintenance of Attendance at Walking Groups: A Longitudinal Multi-Perspective Study of Walkers and Walk Group Leaders

**DOI:** 10.1371/journal.pone.0118754

**Published:** 2015-03-16

**Authors:** Aikaterini Kassavou, Andrew Turner, David P. French

**Affiliations:** 1 Behavioural Science Group, Primary Care Unit, Department of Public Health and Primary Care, Institute of Public Health, University of Cambridge, Cambridge, United Kingdom; 2 Applied Research Centre in Health & Lifestyle Interventions, Coventry University, Coventry, United Kingdom; 3 Manchester Centre for Health Psychology, School of Psychological Sciences, University of Manchester, Manchester, United Kingdom; University of Bath, UNITED KINGDOM

## Abstract

**Background:**

There is good evidence that when people’s needs and expectations regarding behaviour change are met, they are satisfied with that change, and maintain those changes. Despite this, there is a dearth of research on needs and expectations of walkers when initially attending walking groups and whether and how these needs and expectations have been satisfied after a period of attendance. Equally, there is an absence of research on how people who lead these groups understand walkers’ needs and walk leaders’ actions to address them. The present study was aimed at addressing both of these gaps in the research.

**Methods:**

Two preliminary thematic analyses were conducted on face-to-face interviews with (a) eight walkers when they joined walking groups, five of whom were interviewed three months later, and (b) eight walk leaders. A multi-perspective analysis building upon these preliminary analyses identified similarities and differences within the themes that emerged from the interviews with walkers and walk leaders.

**Results:**

Walkers indicated that their main needs and expectations when joining walking groups were achieving long-term social and health benefits. At the follow up interviews, walkers indicated that satisfaction with meeting similar others within the groups was the main reason for continued attendance. Their main source of dissatisfaction was not feeling integrated in the existing walking groups. Walk leaders often acknowledged the same reasons for walkers joining and maintaining attendance at walking. However, they tended to attribute dissatisfaction and drop out to uncontrollable environmental factors and/or walkers’ personalities. Walk leaders reported a lack of efficacy to effectively address walkers’ needs.

**Conclusions:**

Interventions to increase retention of walkers should train walk leaders with the skills to help them modify the underlying psychological factors affecting walkers’ maintenance at walking groups. This should result in greater retention of walkers in walking groups, thereby allowing walkers to receive the long-term social and health benefits of participation in these groups.

## Background

Major health benefits are gained when people initiate and maintain physical activity on a regular basis [1,2]. However, there is a large reduction in participation in physical activity for older people around the retirement period (65–74 years) [3], so many fail to derive most of their potential benefits of physical activity [1].

Walking in groups has been found to increase moderate physical activity among adults, for at least six months after initiation [4]. Walking groups are also becoming very popular in many countries. In England a single organisation that co-ordinates walking groups reported more than 70,000 regular walkers per year [5]. Similarly, in the US one walking scheme reported that almost 400,000 people took part in its walking groups over a two-year period [6]. However, little is known about why people initiate and maintain attendance or drop out of walking groups. Without evidence of the reasons for initiation and maintenance at walking groups, it is difficult to develop walking groups where attendance is better maintained.

### Theoretical Explanation of Initiation and Maintenance at Walking Groups

A central idea in many theories is that a person’s decision to initiate a health behaviour change process is based on their assessment that the benefits afforded by the new behaviour compare favourably to their current situation [7,8]. Further, the same theories propose that maintenance of a health behaviour change is based on people continuing to perceive benefits of the new behaviour, relative to the benefits they initially expected. When the outcomes of this assessment are sufficiently positive, then people tend to maintain the behaviour. When the outcomes of this assessment are not sufficiently desirable, then people tend to cease the behaviour [8,9].

### Evidence for Initiation and Maintenance of Attendance at Walking Groups

Several previous studies have elicited the views of walkers attending existing community groups [10–13]. These studies were all cross-sectional, and used focus groups [10,11], interviews [11,13] and surveys [12,13] to elicit views, which were all analysed using thematic analysis [10,13], content analysis [11] or by assessing the frequencies of responses to questionnaire items [12,13]. Across these studies as a whole, the reasons people give for initiation and maintenance of participation in walking groups were: the social contact, the chance to become more active, the health benefits, the enjoyment of being in the natural environment and the proximity to walks. Moreover, barriers for maintenance were perceived to be: the lack of safety, the lack of confidence to walk, the deterioration in health, the lack of time, joining another activity, the difficulty of walks, the difficulty in accessing the place, the lack of companionship, the bad weather and no specific reason given [10–13].

Efficacy studies evaluating walking group interventions from different countries report similar findings. For example, studies conducted in Australia [14] and the US [15–17] were all cross sectional, and used focus groups [14], multiple methods [15,16] and surveys [17] to elicit views of barriers and facilitators of participation and maintenance in walking group interventions. All of these studies suggested that facilitators for both uptake and maintenance of attendance at walking groups were the health benefits and the social support provided. Barriers for both uptake and maintenance were beliefs about walking capacity and difficulties in accessing the walking place and facilities.

Despite this evidence from previous research, it is all based on cross-sectional snapshots of reported needs, which limits our ability to understand whether and how the factors presented contribute to attendance at walking groups for longer periods of time. To our knowledge, no research has aimed to identify the factors that motivate walkers to join walking groups, whether these factors are the same after a period of time and whether and how these factors support maintained attendance. To address this gap in evidence there is a need for studies of longitudinal designs.

### Evidence on Practises Taken to Facilitate Group Walkers’ Needs and Expectations

The majority of walk leaders are lay people who lead walks voluntarily and have no professional role related to physical activity promotion (e.g. most of them are walkers themselves). Walk leaders receive basic training, which focuses on ensuring that walks are safe, well run and friendly for walkers (e.g. benefits of walking, route planning). They also deal with any issues that arise while walking in groups. Thus, it can be argued that part of their role is to implement practises to meet walkers’ needs and expectations.

There is meta-analytical evidence that walking interventions implementing practises that were tailored to participants’ needs were more efficacious at promoting walking [18]. Given that tailoring interventions to walkers’ needs is important, understanding the steps taken within walking groups to meet walkers’ needs, and views on feasibility and acceptability of these practises seems to be important.

A survey that explored walk leaders’ experiences with their roles showed that walk leaders reported challenges concerning their abilities to provide support to walkers, organisation duties, and role responsibilities [19]. Although previous research has identified walk leaders’ reported barriers and facilitators with their role, no previous research has explored walk leaders’ views about walkers’ needs and their perceived efficacy of their actions to address these needs. To improve our understanding of these issues there is a need for multi-perspective research designs.

### The Present Study

The aims of the present study were: (a) to elicit the needs and expectations from people when joining walking groups; (b) to identify the extent to which these needs and expectations were met three months later, and if not, walkers’ views on why not; (c) to identify how changes in initial needs and expectations might have happened; and (d) to elicit reports of actions taken by walk leaders to address walkers’ needs.

## Methods

This research was approved by Coventry University Ethics Committee before any participant was contacted. All participants were sent an information sheet describing: the aims of the research, participants’ right not to participate in this research or withdraw their data after participation, the interview process (e.g. the use of audio recording), possible benefits, risks and disadvantages of taking part in the research, how data will be used, confidentiality, and an introduction to the research team. All participants gave written consent confirming that they had read the information sheet and agreed to take part in this research.

### Design

Semi-structured interviews were used to gather participants’ views [20]. A longitudinal design was used, with walkers interviewed when joining walking groups and again three months later. A longitudinal design was chosen to capture walkers’ experience, as this evolves through time during their participation in walking groups. A cross sectional design was used for interviews with walk leaders, to capture their accumulated knowledge of walkers’ needs and their practises to address these needs, as they had already lead walking groups for a period of time.

### Participants and Recruitment

All participants above 18 years old were eligible to participate, apart from people with learning disabilities, other medical problems that may have impaired their cognitive abilities, and those with limited facility with the English language.

Participants were recruited and interviewed between July 2009 and March 2010. Walk leaders’ contact details were provided to the research team by a walking scheme run by an urban local authority (Coventry City Council) in the Midlands of England, which was part of the Walking for Health (WfH) scheme. Further, walk leaders who agreed to participate in this research provided the research team with the contact details of walkers who had just joined their walking group. Both walk-leaders and walkers were sent an information sheet by post and asked to participate in a face-to-face interview.

Nine walkers, when they first joined eight different existing walking groups were approached and asked whether they agreed to be interviewed. One person declined, providing work commitments as a reason. Eight walkers agreed to participate in this interview with a mean age of 60.5 years (sd = 3.9). All eight participating walkers were contacted again after a period of three months and asked whether they agree to be interviewed again. Five walkers agreed to participate in the second interviews. All five walkers reported that they had dropped out from the walking groups temporarily. Two of them reported Christmas holidays as a reason and three of them family commitments as reasons for dropping out. The other three walkers declined to participate in the second interview and indicated that they had permanently ceased attending walking groups: one provided the death of a spouse as a reason, the other hospitalisation and the third a lack of time. For information about participating walkers see Table 1.

**Table 1:** Demographic characteristics, reasons for not participating at follow up interview, and how walkers described their attendance at walking groups and walk leaders’ demographic characteristics.

Walkers’ characteristics					Walk leaders characteristics		
Pseudonyms	Gender	Age	Follow up interview	How participants described their attendance at walking groups at follow up	Pseudonyms	Gender	Age
Amelia	Female	56	No	Dropped permanently due to her husband’s death	Oliver	Male	63
Ava	Female	64	No	Dropped permanently due to hospitalisation	Riley	Male	71
Jacob	Male	62	Yes	Dropped out temporarily due to Christmas holidays	Grace	Female	73
Mia	Female	64	Yes	Dropped out temporarily due to Christmas holidays	Jack	Male	73
Lily	Female	63	Yes	Dropped out temporarily due to family commitments	Emily	Female	73
Rudy	Female	54	No	Dropped out temporarily due to lack of time	Sophia	Female	54
Rachael	Female	63	Yes	Dropped out temporarily due to family commitments	Jessica	Female	36
Evie	Female	58	Yes	Dropped out temporarily due to family commitments	Isabella	Female	45
**Mean age (years)**		**60**					**51**

Fourteen walk leaders from fourteen different walking groups were invited to participate. Three of them declined participation: One had dropped out from the walking scheme, one was excluded because he satisfied an exclusion criterion (i.e. medical problem), two participants were not interested in participating and two had not yet established walks when approached. Eight of them agreed to be interviewed (five women and three men) with a mean age of 61 years (sd = 14.4). Most of the walk leaders had long-term experience in leading walking groups and only one had recently been trained. Information about participating walk leaders is provided in Table 1.

In line with the recommendations of Chamberlain and colleagues [21], recruitment of participants was ended when data saturation was achieved. This was satisfied when rich data that were sufficient to answer the research questions were gained. This was done by constant comparison between the categories that emerged from each new interview with those that emerged from earlier interviews. When the last two interviews did not add any new categories to the existing ones, then the data collection ended. Saturation was achieved for interviews with walk leaders and the baseline interviews with walkers but was not fully achieved for the follow up interviews with walkers. All participants were asked about the same topics, however when saturation was achieved in some of the themes, the researcher attempted to explore other themes that emerged at the sequential interviews. Thus participants at the follow up interviews might have been asked to elaborate more on the themes where saturation was not achieved in previous interviews.

The first author conducted and transcribed all interviews, and also conducted the analysis with input from the last author. The authors met to discuss coding and any discrepancies during the process of analysis. The first author had experience with qualitative research, the second author with qualitative methods and group based research and the last author with qualitative methods and physical activity research.

Participants chose the place and time of the interviews. During the interviews only the interviewer and the interviewee were present. Each interview lasted approximately 45 to 90 minutes. All the interviews were audio taped using a digital audio recorder. Following transcription, all information that could lead to participant identification was removed or modified (e.g. using pseudonyms) to retain anonymity.

### Interview Topic Guide

An interview guide was developed by the first author based on relevant literature relating to both theory and research based evidence. The second and third authors commented on the interview guide. At the first set of interviews, walkers were asked to talk about their needs and expectations for joining walking groups. Three months later they were asked about whether their initial needs had been met or not and to elaborate on their reasons for this. Walk leaders were asked to talk about what they believed walkers’ needs were when they join walking groups, and walk leaders’ actions to address these needs.

The period when interviews with walkers took place overlapped with the period when interviews with walk leaders took place. However, the set of interviews with walk leaders finished after the end of the follow up interviews with walkers. This was done so that issues that arose from walkers’ interviews could be followed up at some interviews with walk leaders. The researcher also aimed to follow up themes that arose from walkers’ interviews with walk leaders from all walking groups, i.e. not only with the leader from the group that a specific walker attended.

### Analysis

Two preliminary thematic analyses were conducted, one with the cross sectional interviews with walk leaders and one with the longitudinal interviews with walkers. Thematic analysis is a method of systematically detecting, categorizing, interpreting and reporting patterns or themes within raw data [22]. For the cross sectional analysis, the process described by Braun and Clarke [22] was followed. For the longitudinal thematic analysis, each participant’s baseline and follow up interviews were analysed as case studies. All interviews were analysed separately, then themes were collated across all longitudinal interviews. The themes produced described the latent meaning regarding experiences with walking groups shared by most participants from initiation to three months participation. Where there were themes that were unique to individual participants from the case studies analyses, these were treated as subthemes in the aggregated analysis.

The themes identified from separate analyses with walkers and walk leaders were then integrated in a longitudinal multi-perspective thematic analysis. The aim of this analysis was to identify walkers’ and walk-leaders’ perception about walkers’ needs when joining walking groups and whether these were satisfied three months later [23]. Themes identified in the interviews with walk leaders were mapped onto those of the interviews with walkers. These broader themes are presented in terms of similarities and differences between walk leaders’ and walkers’ perspectives. Where themes did not fit comfortably within broader themes, they were investigated separately.

Broader themes were then explored in terms of whether they described separate phenomena at a specific time point, such as walkers’ needs, or the experiences or changes across time, such as processes to meet these needs. A more detailed description of the process of analysis is described in details provided in Supplementary Information.

## Findings

Three themes across walkers and walk leaders were identified by the longitudinal multi-perspective thematic analysis: social connectedness and healthy lifestyle, perception of control and actions to address walkers’ needs.

### Social Connectedness and Healthy Lifestyle

Both walkers and walk leaders acknowledged that walkers joined walking groups to feel socially connected and to gain a healthy lifestyle. When they joined, walkers tended to compare their present unsatisfactory social or health situation with the one they desired to achieve.

“Chatting to people making friends and hopefully getting a social circle which I don’t have at the moment” (walker Ava, 64 years old, baseline interview).

“But the walk was a next step up from everything else I was doing, actually I thought I’ve got to start improving my fitness. So I wanted to lose weight as well” (walker Jacob, 62 years old, follow up interview).

“Well I think they are expecting to get fit and healthy, but apart from that the bonus is that they make new friends, which is good” (walk leader Riley, 71 years old).

Walkers particularly tended to emphasize more the health benefits they expected to gain from joining walking groups, rather than the social aspects.

“It was the fitness part cause I am looking in improving my health but the social side was an added bonus, it was, I am glad, I am glad I joined it” (walker Amelia, 56 years old, baseline interview).

“That again is good exercise and I can move, so why not, and would be fun, so if I can get fit and have fun at the same time why not, so I shall see how that goes” (walker Ava, 64 years old, baseline interview).

When walkers first attended walking groups, they engaged in social comparisons, triggered by meeting other people in the walking group. A suitable model for social comparison was a person who was perceived to be similar to walkers, in terms of similar needs to themselves and similar expectations for the means to meet these needs (e.g. feel socially connected via social interaction).

“I was expecting some people to be in the same boat as me. I thought they would be people that they are looking for something to do with the social aspect same as me” (walker Amelia, 56 years old, baseline interview).

Walkers who participated in the follow up interviews three months later, were people who had dropped out from walking groups temporarily (i.e. had missed some sessions). Some of them expressed an intention to continue attendance and some others expressed an intention to drop out from that walking group and/or join another group or activity.

The walkers who expressed their intention to maintain attendance at walking groups indicated that satisfaction of their initial needs was their main motivation to do so. These walkers tended to focus their description on the social feedback they were getting from other people in the group and the physical feedback associated with health or fitness benefits of walking. For example, they tended to describe the other members of the groups as being pleasant and friendly, and to describe the short-term goal achievements (e.g. walking distance) or subjective feelings after walking.

“I suppose if the people weren’t nice I wouldn’t go so I suppose the people are the main reason that you go because otherwise you join another group or you wouldn’t go” (walker Rachel, 63 years old, follow up interview).

“You do feel healthier, it’s like you go to the gym and you got to work out of it, think for walking as a gym […] and you can feel the difference when you have done the walk, it’s three miles, takes an hour, you can feel different, you start feeling different, you start to feel better on yourself, your well-being” (walker Jacob, 62 years old, follow up interview).

“It’s very nice and encouraging to see how their health improves. They can do things that they couldn’t do before. People come down and say I can only do half a lap, I can’t do anymore. After a few weeks they are doing a lap, and then it goes to two laps, and people are amazed that they can do it […] it’s just a bunch of friends” (walk leader Riley, 71 years old).

“You know, they enjoy the same things. You meet some nice people in same the position as you are” (walk leader Riley, 71 years old, follow up interview).

The walkers who expressed an intention to drop out from that walking group and/or join another group or activity mentioned their dissatisfaction with meeting their initial needs. Where walkers’ needs for social connectedness were not achieved, some walkers focused their descriptions more on the expected health benefits. However, these walkers still acknowledged that their social needs remained an important motivation for continuous participation at walking groups.

“I mean originally, we thought the social thing would be good […] but it’s not that we need more social life, we’ve got more than enough, so we really do go just for the walking” (walker Mia, 64 years old, follow up interview).

Some walkers who intended to drop out tended to attribute dissatisfaction with social aspects of walking groups to other people having an established routine, due to frequent attendance over the longer term.

“I wouldn’t say that people are as friendly as I hoped they would be maybe it’s because I don’t, the others they will be going for a long time […] but no it doesn’t bother me” (walker Mia, 64 years old, follow up interview).

Walk leaders also acknowledged that dissatisfaction of walkers’ initial needs were reasons for walkers to cease participation from walking groups. It might be the case that walk leaders tended to report their accumulated knowledge from their experience with leading walking groups or could not distinguish that the processes walkers go through might differ from initiation to maintenance at walking groups. It could also be that walk leaders did not like to elaborate more on the reasons walkers stop attending walking groups, as this would not have been in favour to their co-ordinating scheme. It should also be noted that walk leaders were approached first by the co-ordinating scheme and then were referred to the researchers. A selection bias might have been introduced, so that only walk leaders with positive experiences with the scheme were selected for this study.

Also, it might be the case that at initiation walkers emphasised their need to gain health benefits more than social benefits. It might be the case that walkers did not like to talk more about what made it difficult to them to continue participation in walking groups. Instead, when asked to evaluate their experiences with walking groups, they might have selectively reported the possible health benefits they would get. This could be due to socially desirable reasons: not to sound unpleasant, not to be unfair to walk leaders or to present themselves as someone who is more interested in getting healthier rather than having unmet social needs.

### Perception of Control

Both walkers and walk leaders acknowledged that a perception of control over satisfying their initial needs via walking groups, was important for initiation of attendance at walking groups and that the other members of the group could be a source of feelings of efficacy for walkers. Verbal persuasion or vicarious information from people who had themselves participated in walking groups seemed to be an effective method for motivating walkers to join walking groups.

“It was only because a friend at the church said “do you fancy doing that?” you know, we go to park on Saturday morning, we do a walk round there, if you fancy doing some walking, I thought that seems like a good idea, so that’s how that started really” (walker Mia, 64 years old, baseline interview).

Walkers who participated in the follow up interviews acknowledged that perception of control seemed to be important for maintained attendance at walking groups. It seemed that walkers assessed both abilities for achieving social connectedness and fitness within walking groups, by comparing themselves with similar walkers in the group. When the outcome of this assessment was positive, then walkers tended to report that they were satisfied with their participation in walking groups.

“Well it gives you confidence. Well you know if they can do it why can’t I?” (walker Evie, 58 years old, follow up interview).

When the outcome of this assessment was negative, then walkers tended to report that they were not satisfied with their participation in walking groups and they intended to cease participation from that group.

“If it would still be like the cold shoulder might think of blow this thing what if I do it on my own, you know with my wife, go around together and that would be it, but I don’t think it will come to that, I have a feeling that it will not come to that” (walker Jacob, 62 years old, follow up interview).

Some walk leaders acknowledged that other members of the groups are a key source of satisfaction and efficacy for walkers, however walk leaders seem not to fully report/acknowledge walkers’ perspectives about the importance of meeting other walkers with similar needs and expectations to themselves.

“It makes you feel you are a better person. It gives you confidence that you’re able to do these things. Because everybody is going into the unknown, but if somebody has been there before they can help” (walk leader Riley, 71 years old, follow up interview).

Some walk leaders also reported some other factors as important for walkers’ maintenance of attendance at walking groups, notably walkers’ willingness to participate in walking groups or environmental factors (e.g. weather).

“But over the year, when the weather changes, people drop out, some come back, some don’t” (walk leader Riley, 71 years old).

Walk leaders’ estimates of their ability to facilitate walkers’ needs seemed to depend upon their perceptions of the factors that influence walkers’ maintenance at walking groups. Those walk leaders who perceived that walkers’ participation at walking groups was not greatly influenced by their actions, reported low efficacy to address walkers’ needs.

“I mean he [the walk leader] can do a bit with his personality. Some people they’ve got things to do, so they drop out but there is no solution to that one. I’ve never thought about it, but I can’t see anybody solving it. Really it is just a matter whether the newcomers can then think it is worthwhile, you see how could you convince them that it is worthwhile? You know you have to strike the balance and it is impossible to achieve some times. Oh dear it is difficult” (walk leader Jack, 73 years old).

### Reports of Actions Taken by Walk Leaders to Address Walkers’ Needs

There were only a few walk leaders who reported several actions that they take to support walkers’ integration into groups and increases in walking when walkers join and/or maintain attendance at walking groups. Some of these actions were: assessing and tailoring the walk to walkers’ needs, introducing walkers to existing walkers, explaining the benefits of walking and the type of walking the group does, providing walkers with pedometers, providing walkers with feedback on behaviour, providing rewards, making telephone calls for supporting continued attendance, and encouraging walkers to increase their walking.

“The encouragement to get people to speed up so they are getting the maximum benefit that they can from the walk […] to looked pleased to see them […] to introduce them to the group […] have to be respectful of how they wanted the walk to be and take the time to build the relationship with them and trust with them” (walk leader Sophia, 54 years old).

“My duties are, make sure they’ve done the health questionnaire, make sure I have first name knowledge and I know the history, where they are coming from and what their fears are […] we need to know why they are there […] they were using the pedometer to say how far they walk “that’s good for you” and then we were offering them something good at the end of it […] and I ring everybody so Saturday walk and so, come and join […] one person can talk about what benefits you have from this one […] explain what benefits you can get” (walk leader Jessica, 36 years old).

However, walk leaders attributed successful and continuous attendance at walking groups to uncontrollable factors (e.g. luck) or to walkers’ characteristics (e.g. pleasant people) and not to their or walkers’ actions.

“I don’t know it’s something that we are lucky, all the long term walkers we’ve got are a nice group of people, so they are open and they are accepting to others you know” (walk leader Jessica, 36 years old).

On the other hand, the walkers who expressed their intention to continue attendance at walking groups expressed their satisfaction with walk leaders’ actions when they joined and maintained participation at walking groups. These actions were related to walk leaders taking into consideration walkers’ needs and expectations and tailoring their recommendations to these needs.

“I think it’s nice they are taking interest in the people that are on their walks. Well they try to meet the expectations of other people […] it’s makes you feel like they know everybody’s needs […] they make it easy for you to ask things […] when I got the phone call from her we are meeting somewhere else […] it was nice to know that she recognized me as a new starter” (walker Amelia, 56 years old, baseline interview).

“It looks like he arranges some meals and it’s like you meet at that place, sit down and have a natural meal and it’s the social side of it” (walker Mia, 64 years old, follow up interview)

“He is very encouraging, he is very nice man, of course because he is enthusiastic you feel that you don’t let him down” (walker Lily, 63 years old, follow up interview).

“She is very good anyway and she is friendly and at the minute she is trying to sort out different walk […] she does things like that you know gives you the choice, you know she is not “right we go so and so and that’s it”, no we discuss where we are going so that everybody is happy sort of thing” (walker Evie, 58 years old, follow up interview).

However, the walkers who expressed their intentions to drop out from walking groups did not seem to acknowledge actions within walking groups, aimed at supporting their social integration or healthy lifestyle.

“Depends on who you are walking with and who is sitting next to you during coffee time after walking […] you are only talking to whoever pops in with you, at your speed […] not really, I wouldn’t say welcome really, you know, a greeting, a smile, a hello and shaking hands with the man, that sort of things, but if you don’t get any of those, you think grrr terrible, isn’t it?” (walker Jacob, 62 years old, follow up interview).

In these cases, a possible explanation could be that walk leaders reported their roles as walk leaders and not their actions to address walkers’ needs, since walkers seemed generally not to acknowledge that their leaders’ actions were effective at integrating new walkers to the walking group.

## Discussion

### Principal Findings

The decision to join a walking group was facilitated by walkers’ needs to be both socially connected and healthy. It appeared that walkers engaged in comparison processes with similar others in the group in order to make sense of their experiences and appraisals of their ability to emotionally and proactively cope with challenges during the first weeks of attendance at walking groups. When these processes of social comparison provided walkers with feelings of efficacy to cope with challenges while making changes, then walkers’ initial needs were satisfied. This feeling of satisfaction was associated with meeting the other people in the group. Further, to maintain this feeling of satisfaction, walkers tended to maintain participation at the walking group in which these other people were present.

Walk leaders also acknowledged that social connectedness and healthy lifestyle were the main reason for walkers to join walking groups and that satisfaction with meeting other members of the group was the most important reason for walkers’ maintenance at walking groups. However, walk leaders appeared not to view these processes as important sources of efficacy for walkers to continue attendance. Walk leaders seemed to also report some other factors important for walkers’ maintenance at walking groups. These were relevant to walkers’ willingness, environmental factors, or their actions. Walk leaders’ perceptions of the factors that influence walkers’ maintenance at walking groups seemed to have an effect on their perception of their ability to facilitate walkers’ needs. Those walk leaders who perceived that walkers’ participation was less influenced by their actions reported low efficacy to address walkers’ needs.

### Strengths and Weaknesses of the Study

The sample employed for this study consisted of walkers and walk leaders recruited from a single urban location in England. Moreover, the sample recruited for this study mainly consisted of older women. Thus these findings should be treated with caution when generalising to other non-urban areas and demographic groups. Despite this, the majority of people who participate in WfH group walks in England are older women [24], so it is reasonable to suppose that the findings of this study could provide a fairly representative picture of the other walking groups within England or other Western countries.

Irrespective of this, the primary aim of this qualitative study was not to recruit a representative sample and generalize findings to the whole population, but to generate the most relevant information to answer the research question and apply recommendations tailored to the included population.

A strength of this study was that the majority of potential participants agreed to participate in the interviews, with only a minority of walkers not willing to be followed up (three out of eight). The three people who declined participation in the follow up interview had ceased attending walking groups and were unwilling to be interviewed. Although their views would be valuable for this study, the problem of dropouts being unwilling to participate in research is well known [25]. It should also be noted that the five walkers who participated in the follow up interview had also temporarily dropped out from walking groups, but were willing to talk about the challenges that they had faced during the past three months. It might be expected that the views of those who continue to use a service and those who do not would differ substantially.

To our knowledge this is the first study that has explored both the needs and expectations of people when they joined walking groups and whether these needs and expectations had been met three months later. This study not only identified needs and expectation at two time points, but also the psychological processes that facilitated or challenged the satisfaction of these needs during this period of time. However, it should be mentioned that saturation was not achieved for follow up interviews with walkers.

An important strength of this study is its multi-perspective approach, which provided the opportunity to generate a better understanding of walkers’ and walk leaders’ needs, and to explore how walk leaders understand walkers’ needs and what actions they take to address these needs. This is an important approach especially when aiming at evaluating service effectiveness and generating suggestions for improvement.

### What Does this Study Add to the Existing Literature?

Previous research has identified motivation at initiation [11,14,26] and maintenance of attendance [10,12,15] and barriers to participation [11,15,27] at walking groups. In summary, previous research suggested that people initiated and maintained attendance at walking groups in order to gain social and health benefits and to enjoy the natural environments. By contrast, challenges identified were concerning: the lack of safety, time, facilities, accessibility, confidence, the weather, and health problems.

Previous research did not however explore the stability of motivations and challenges at initiation and at three months maintenance of attendance at walking groups. Moreover this previous research did not explore the psychological process underlying walkers’ motivations at initiation and three months maintenance of attendance. The present research identified that satisfaction of walkers’ initial needs was an important factor for maintenance at walking groups and that walkers’ reporting of initial needs changed over time. For example, even though walkers reported that they joined walking groups to gain a healthy lifestyle, at the follow up interview they reported that social connectedness seemed to be more salient motivation for their participation at a particular walking group. The longitudinal design of the present study facilitated the identification of underlying psychological process over time, which could not be done by previous research employing cross-sectional designs.

Previous studies have not used a multi-perspective approach to investigate walkers’ reasons for participating in walking groups. Multi-perspective interviews can show different facets of walkers’ needs and ways to address them based on the role that each participant has. The present research identified that walkers perceived other members of the group as important source of both satisfaction and efficacy. It also showed that walkers engaged in social comparisons with other walkers in the group to assess their progress towards social integration and healthy lifestyle. However, even though walk leaders acknowledged the importance of other walkers, they did not seem to acknowledge that this was the process that walkers went through when they joined walking groups and that this process was important for their maintenance.

Moreover, the multi-perspective approach provided different perspectives on the services, which could contribute to formulating tailored recommendations for improving these services. This research identified that although some walk leaders reported several actions to address walkers’ needs, walkers who expressed their intention to drop out from walking groups did not report that these were effective at promoting maintenance at walking groups. For example, walkers were dissatisfied when their needs to socialise with similar others were not met, and walk leaders agreed with this point of view. Despite this, walk leaders attributed difficulties walkers faced in joining new groups as being beyond their control and their actions, instead of walk leaders tending to report low efficacy to address these difficulties. The lack of reported efficacy and/or effective strategies to address walkers’ needs also suggested the need for further actions that might be taken to improve the current situation, such as training. These strategies can be drawn from those walk leaders’ actions who effectively addressed walkers’ needs and relevant literature.

### Implications for Policy and Practise

The findings of this study provide some useful insights on the role of walkers’ needs and expectations to support maintenance of attendance during the first three months after joining a walking group. These findings have been supported by a longitudinal quantitative study, building on the findings of the present study, which found that satisfaction with social connectedness and health benefits predicted maintenance of attendance at walking groups [28].

Walking groups can promote moderate physical activity in the long term, i.e. more than six months [4] and are potentially inexpensive to implement. Given this, if adequate training is provided to lay leaders [29], it becomes essential to determine how best to ensure that walkers experience social connectedness and health benefits. Thus, assessing walkers’ needs and expectations when they join walking groups and implementing practises to address these needs seems to be important for maintained attendance at walking groups.

Interventions are likely to be more effective if they target the underlying psychological processes that produce walkers’ desired outcomes [30]. This study suggested that satisfaction with social connectedness with similar others was an important underlying psychological processes for walkers' to gain efficacy to cope with challenges and maintain attendance at walking groups. Some techniques could be to allocate people with similar needs and expectations to the same walking groups, especially new members, to support communication within members of the groups via social and walking related activities, social modelling, and discussion with more established members of the group about benefits of walking groups, the process followed to successfully achieve these benefits, and offer advice tailored to walkers’ needs. Another effective technique to support maintenance of attendance at walking groups could be to refer walkers to those walking places where contextual factors produce experiences in line with their expectations, e.g. lap walks around a sports track are not well suited to prompting social interaction [31,32].

Providing training to walk leaders with techniques to promote walking within walking groups might be a useful way of helping walkers to achieve social connectedness. However, in doing so, consideration should be given to walk leaders’ perceptions of how changes in walkers occur and the actions needed to address walkers’ needs. For example, the present study showed that some walk leaders were apparent not aware of the processes underlying walkers’ social connectedness and satisfaction, and how these influence uptake and maintenance of attendance at walking groups, or how walk leaders’ actions facilitate or not these processes.

In conclusion, to date no research has been done to identify whether and if the techniques mentioned above are effective at increasing physical activity, group connectedness or any other outcomes such as quality of life in efficacy or effectiveness studies within lay-led, community based walking schemes. Future research should identify what type of training is effective for promoting those behaviours of walk leaders that are likely to result in walker satisfaction, what strategies are effective at promoting specific outcomes for walkers, and aim to identify potential contextual influences. Future research might also usefully investigate whether training interventions for walk leaders are effective at increasing satisfaction and retention of walkers, and are cost effective.

## Supporting Information

S1 File Process of analysis. (PDF)
