# Supplementary material for: The Role of Walkers’ Needs and Expectations in Supporting Maintenance of Attendance at Walking Groups: A Longitudinal Multi-Perspective Study of Walkers and Walk Group Leaders
Source: PLoS One. 2015 Mar 16;10(3):e0118754. doi: 10.1371/journal.pone.0118754 (PMC4361621; doi:10.1371/journal.pone.0118754)
Supplement: S1 File — (PDF) [file pone.0118754.s001.pdf]

Process of longitudinal multi-perspective analysis

Interview transcripts were subjected to thematic analysis (Braun & Clarke 2006).

Initially, two separate thematic analyses were conducted, one cross-sectional and one longitudinal. These analyses aimed at identifying themes across participants of the same group. The cross sectional analyses looked at identifying themes within the specific point of time (i.e. when the narratives occurred), whereas the longitudinal analysis looked at identifying themes along a period of time (i.e. when experience with the phenomenon under study was involved). Based on these analyses, a longitudinal multi-perspective analysis explored themes across participants of different groups and across time.

Detailed description of the process of analyses followed at this study is described below:

Cross sectional thematic analysis

One cross-sectional thematic analysis was conducted with the interviews with walk leaders. Below there is a description of the phases involved in this analysis:

a) After the interview, the researcher noted thoughts and descriptions related to the interview process (e.g. body language, practical challenges etc) and transcribed the interviews.
b) The researcher read all transcripts line by line so that she got familiar with the information included in the transcript. At the same time the researcher listened to the recordings and made any corrections to the transcripts. The transcripts were read as many times as needed for the researcher to feel familiar with the data. While listening to the recordings the researcher highlighted those words that were used by participants in a meaningful way, for example words that were used metaphorically or had emotional value for participants and made relevant notes at the margin of the transcripts. This was done in order to get more insight into how participants made sense of their experiences. These notes were used to draw a wide picture of what is in the data and guide the next phase of analysis.

c) Ideas important for describing each participant's experiences were identified and written in a form of a list, separate for each interview. These ideas, which are called codes, are meaningful units of raw information and provide a detailed description of participants' experience. Codes could refer to a manifest content of the data (when something was directly observable) or more latent level (when something was implicitly referred to: see b). Each interview was coded separately in order to identify themes within participants of the same group. Following this, the researcher produced for each interview a table with codes and extracts of raw data. An attempt was made for each interview to be given equal attention.

d) When all interviews were coded separately, the researcher collated codes across interviews with the aim to identify themes across participants of the same group. A list of different codes was produced with codes identified across data set.

e) The main themes of the analysis were produced. Themes are broader concepts that consist of collated and combined codes, represent a patterned response and shape data in a meaningful units, relevant to research topic. Themes usually focus on the shared meaning of the codes produced. During this stage different codes were combined together to form a theme. The researcher re-read transcripts several times and went backwards and forwards (between raw data, codes and themes), until she felt confident enough that different codes, could collate together and form a theme. Visual representation (i.e. map and graph with themes) was made to facilitate researcher's comprehension of the whole data set.

f) The researcher went backwards and forwards between raw data, codes and themes and tried to identify those themes that form a coherent pattern and those that do not. When a coherent theme was identified, the researcher re-reads the transcripts to make sure that themes could be generated to the entire data set. To facilitate this, the researcher ticked in a table the frequency that a theme was identified in transcripts. During this process different themes were generated in broader themes. The researcher moved several times between raw data, codes and themes until she feels that saturation had been achieved. There was an attempt so that the themes produced describe the latent meaning shared by most participants' experience with walking groups. In the second case, when a theme could not form a coherent pattern, codes forming the theme were re-defined and attempt was made so that they could be incorporated to existing themes (e.g. as subthemes) or form another new theme.

g) Themes and subthemes produced from above stages were labelled, defined and examples were indicated. Different dimensions of the same phenomenon were apparent at this phase. Themes illustrated manifest and latent features of the same phenomenon across participants of the same group, subthemes illustrated manifest and latent features that could include as well some themes from the within participant analysis (see a,b,c).

Longitudinal thematic analysis

One longitudinal thematic analysis on the baseline and follow up interviews with walkers was done. The process of this analysis is described below:

a) All baseline interviews were analysed via a cross-sectional thematic analysis as this is described above.
b) Before each follow up interview, the interviewer listened to and read transcripts of each participant's baseline interview. This was done so that the interviewer could follow-up with each participant issues that were mentioned at the baseline interviews and identify and explore any new experiences and views, which had not been mentioned at baseline interviews.
c) After each follow up interview, the interviewer analysed each participant's baseline and follow-up interviews with the aim to identify themes within participants across time. The aim was to explore whether, how and for what reasons particular participant's experiences of and views about initial needs and expectations had changed over the period of three months.
d) When all interviews were analysed separately, the researcher collated themes across longitudinal interviews. The researcher looked for similarities and differences between the themes that emerged from within analyses (see b). The aim was to identify themes across participants and across time. The themes produced described the latent meaning shared by most participants' experiences with walking groups across time. Subthemes also included themes from the within participant across time phase (see b), in order to provide contextual information to themes.

Longitudinal multi-perspective thematic analysis

One longitudinal multi-perspective analysis was conducted based on the cross sectional analyses and longitudinal analysis described above. The aim of the longitudinal multi-perspective analysis was to identify themes across groups of participants and across time.

The process of this analysis is described below:

a) The researcher looked for similarities and differences between the themes from the longitudinal analysis with walkers and the cross sectional analyses with walk leaders. There was an attempt to explore similarities and differences of identified themes between groups.
b) The researcher produced a list of themes. There was an attempt to map the themes identified in the interviews with walk leader to those of the interviews with walkers. Similar themes across groups were collated to broader themes. Different themes were also collated to broader themes and presented to explain the context of the broader similar themes (i.e. role influences: walk leader *vs* walkers). Themes that could not be collated to broader themes, were investigated separately, were re-defined and attempt was made to be incorporated to the broader similar or different broader themes.
c) Broader themes were then explored in terms of whether they described separate phenomena at a specific time point or processes across time. This was decided on whether broader themes explained a phenomenon (e.g. needs) or the experience/changes of a phenomenon across time (e.g. process to meet these needs).
